# Shocks in the Very Local Interstellar Medium

**DOI:** 10.1007/s11214-022-00893-4

**Published:** 2022-05-09

**Authors:** P. Mostafavi, L. F. Burlaga, I. H. Cairns, S. A. Fuselier, F. Fraternale, D. A. Gurnett, T. K. Kim, W. S. Kurth, N. V. Pogorelov, E. Provornikova, J. D. Richardson, D. L. Turner, G. P. Zank

**Affiliations:** 1 Johns Hopkins Applied Physics Laboratory, Laurel, MD 20723 USA; 2 NASA Goddard Space Flight Center, Code 673, Greenbelt, MD 20771 USA; 3 School of Physics, University of Sydney, Sydney, NSW 2006 Australia; 4 Southwest Research Institute, P.O. Drawer 28510, San Antonio, TX 78228 USA; 5 Department of Physics and Astronomy, University of Texas at San Antonio, San Antonio, TX 78249 USA; 6 Center for Space Plasma and Aeronomic Research (CSPAR), University of Alabama in Huntsville, Huntsville, AL 35805 USA; 7 Department of Physics and Astronomy, University of Iowa, Iowa City, IA 52242 USA; 8 Department of Space Science, University of Alabama in Huntsville, Huntsville, AL 35805 USA; 9 Kavli Institute for Astrophysics and Space Research, Cambridge, MA USA; 10 Department of Physics, Massachusetts Institute of Technology, Cambridge, MA USA

**Keywords:** Heliosphere, Interstellar medium, Shock waves

## Abstract

Large-scale disturbances generated by the Sun’s dynamics first propagate through the heliosphere, influence the heliosphere’s outer boundaries, and then traverse and modify the very local interstellar medium (VLISM). The existence of shocks in the VLISM was initially suggested by Voyager observations of the 2-3 kHz radio emissions in the heliosphere. A couple of decades later, both Voyagers crossed the definitive edge of our heliosphere and became the first ever spacecraft to sample interstellar space. Since Voyager 1’s entrance into the VLISM, it sampled electron plasma oscillation events that indirectly measure the medium’s density, increasing as it moves further away from the heliopause. Some of the observed electron oscillation events in the VLISM were associated with the local heliospheric shock waves. The observed VLISM shocks were very different than heliospheric shocks. They were very weak and broad, and the usual dissipation via wave-particle interactions could not explain their structure. Estimates of the dissipation associated with the collisionality show that collisions can determine the VLISM shock structure. According to theory and models, the existence of a bow shock or wave in front of our heliosphere is still an open question as there are no direct observations yet. This paper reviews the outstanding observations recently made by the Voyager 1 and 2 spacecraft, and our current understanding of the properties of shocks/waves in the VLISM. We present some of the most exciting open questions related to the VLISM and shock waves that should be addressed in the future.

## Introduction

The Voyager spacecraft have made many discoveries about shock waves since their launch in 1977. They revealed the essential changes in the shock properties as they propagate from the inner heliosphere into the pickup ion (PUI)-mediated outer heliosphere and further into interstellar space. Over the past few decades, various theoretical and numerical models studied and simulated the properties of shock waves and their propagation into the outer heliosphere and beyond. They showed that heliospheric shock waves generated near the Sun propagate in the heliosphere, interact with the heliosphere’s boundaries, and finally, some fraction of them are transmitted into the VLISM (e.g., Barnes 1993; Pogorelov 1995; Story and Zank 1995; Washimi et al. 2011; Borovikov et al. 2012; Pogorelov et al. 2013; Provornikova et al. 2013; Fermo et al. 2015). Through the analysis of intense radio emissions detected in the supersonic solar wind (SW) by Voyagers, Gurnett (1995) first showed that their origin was associated with the propagation of heliospheric shocks beyond the heliopause (HP).

Voyager 1 (*V1*) crossed the HP on August 25, 2012, at 121.6 AU, and since then it has occasionally detected electron plasma oscillation events and sudden jumps of the interstellar magnetic field. Some of these events are believed to be heliospheric shocks propagating into the VLISM (Burlaga et al. 2013a; Richardson et al. 2017). From 2012 to 2021, *V1* identified two shocks and two pressure fronts from magnetic field data (Burlaga et al. 2021). While the two shocks were associated with the electron plasma oscillations detected by the *V1* plasma wave instrument (PWS), two pressure fronts were not concurrent with any plasma oscillations. Shocks in the VLISM are weak and far broader than similar shocks in the heliosphere (Burlaga et al. 2013a). Mostafavi and Zank (2018b) suggested that the thickness of the weak VLISM shock structure, unlike the heliospheric shocks, is determined by thermal particle collisions and is not mediated by wave-particle interactions. In general, Voyager’s observations showed that our knowledge of the VLISM and shocks/pressure waves in it is limited and needs to be expanded extensively based on different mediums and underlying physics.

It was generally known that the SW–LISM interaction creates a collisionless shock and a tangential discontinuity, namely the heliospheric termination shock (HTS) and the HP. An additional bow shock or wave can form in the LISM in front of the HP, depending on the sub or supersonic nature of the relative motion of the heliosphere with respect to interstellar space (Parker 1961; Baranov and Malama 1993; Pauls et al. 1995; Zank et al. 1996b; Williams et al. 1997; Zank and Pauls 1997; Zieger et al. 2013). In addition, a plasma depletion layer (PDL) is expected in front of the HP (Fuselier and Cairns 2013; Cairns and Fuselier 2018). Even though the existence of the HTS has been established by the Voyager crossing (Richardson 2008), questions regarding the bow shock or wave presence and its structure are still open. This is because the properties of the VLISM are very poorly constrained.

This chapter presents an overview of the shock waves, starting briefly from the source of the observed radio emissions in the heliosphere to the possible bow shock or bow wave in the interstellar space with a particular focus on shocks in the very local interstellar medium (VLISM). First, the history of observed heliospheric radio emissions by Voyager probes in the 80s and 90s and their sources is presented in Sect. 2. Possible sources of the observed electron plasma oscillation events in the VLISM by Voyager are then discussed in Sect. 3. We show that electron plasma oscillations are an excellent indicator for estimating the VLISM density profile. Furthermore, this section summarizes the various theories explaining the reason for the continued increasing plasma density immediately beyond the HP. Then, we focus on shocks and pressure fronts in the VLISM (Sect. 4) by analyzing the Voyager observations. We review different models showing the propagation of shocks from the heliosphere into interstellar space. Later in this section, we discuss the collisionality of the VLISM and its effect on shock structure. We briefly discuss turbulence related to the observed VLISM shocks. In Sect. 5, we discuss the deceleration of the VLISM plasma due to its interaction with the heliosphere and the possibility that either a bow shock or bow wave may form depending on the parameters of the VLISM. We present different models and theories about the presence of bow shock or wave in the ISM. Finally, Sect. 6 completes this chapter with some present open questions related to the VLISM and shock waves that need to be addressed in future studies and future dedicated missions.

## Heliospheric Radio Emissions

**Fig. 1:**
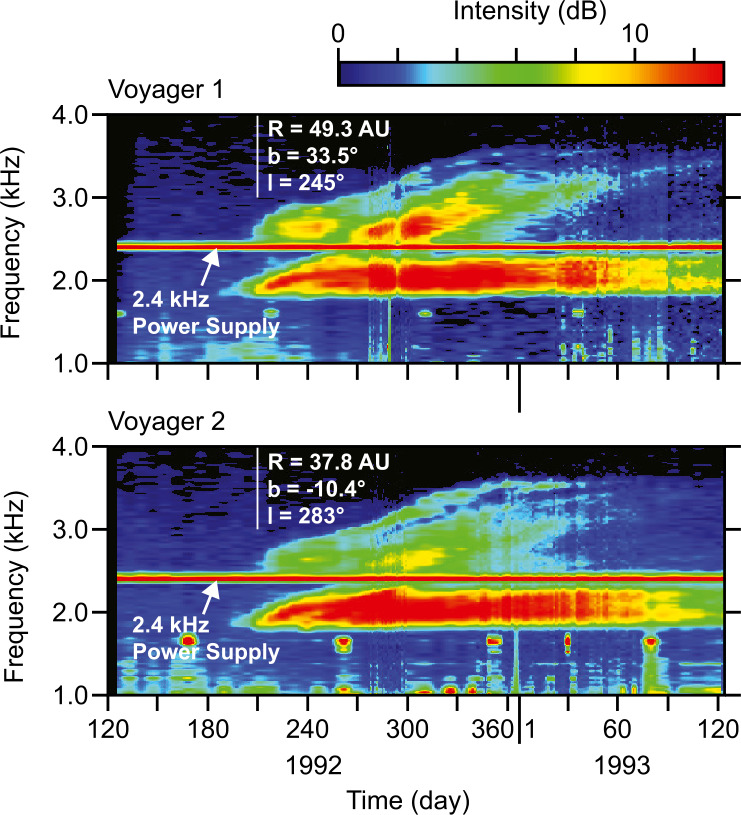
Frequency-time spectrograms show the strong 2-3 kHz radio emissions detected by Voyagers 1 and 2 in 1992-93. Note the increasing frequencies of various components of the emission, which are similar at the two spacecraft. The heliospheric radial distance, R, in AU, and the solar ecliptic latitude and longitude in degrees are shown to the right of the vertical white lines, which mark the points at which these coordinates were determined. This figure is adapted from Gurnett et al. (1993)

This section describes the first indirect observations of heliospheric shock propagation beyond the HP. In 1983-84, the *V1* and *V2* PWS instruments detected an unusual radio emission with slowly rising frequencies in the range from about 2 to 3 kHz. The events started on day 242, 1983, at a radial distance of 17.2 AU for *V1* and 12.4 AU for *V2* (Kurth et al. 1984). The emissions were unusual in that they started with comparable intensities almost simultaneously at both spacecraft, even though the two spacecraft were separated by nearly 10 AU. The close similarity of the frequency spectrum at such a considerable separation distance clearly identified the emission as remotely generated electromagnetic radiation (i.e., radio emissions) and not locally generated electrostatic waves. Although several possible sources were considered, including (1) planetary, (2) heliospheric, and (3) astrophysical, the origin of the radio emission was unknown. About nine years later, another even more extreme 2-3 kHz radio emission event was detected by both Voyagers (Gurnett et al. 1993). Frequency-time spectrograms of the radio emission intensities associated with this event are shown in Fig. 1. As can be seen, the radio emission consisted of several distinct components rising slowly in frequency from about 2.0 kHz to as much as 3.6 kHz on a time scale of about half a year. Again, the intensities at the two spacecraft were similar, in spite of their 44 AU separation. From this close similarity, it was concluded that the source must be located at a distance considerably greater than 44 AU. Using 44 AU as the minimum distance, the total radiated power was estimated to be much greater than any known planetary radio source, suggesting that the radiation was either from a distant heliospheric or astrophysical source.

**Fig. 2:**
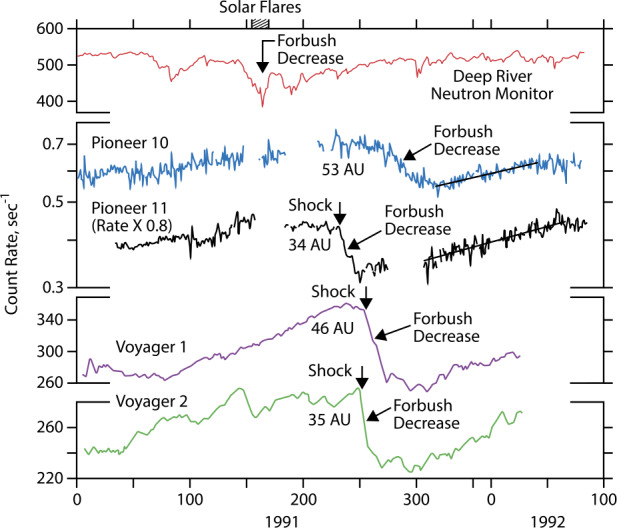
The top panel shows the deep Forbush decrease in the Deep River Neutron Monitor counting rate caused by the intense May 25 to June 15, 1991, solar events. The bottom four curves show the counting rates of energetic charged particle detectors on the Pioneer 10 and 11 and Voyager 1 and 2 spacecraft at various distances from the Sun. This figure is adapted from Gurnett et al. (1993)

**Fig. 3:**
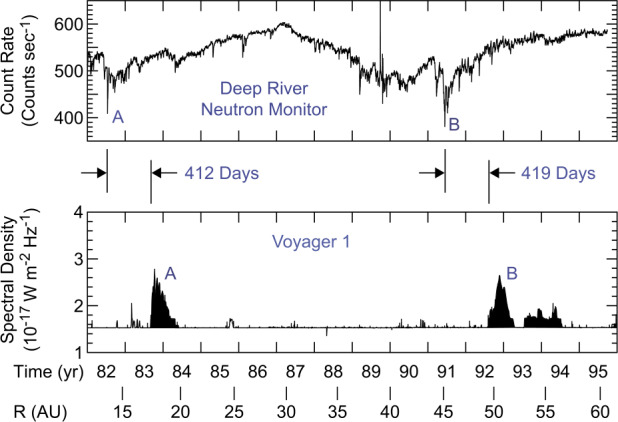
These two panels show the relative timing of the very deep Forbush decreases caused by the intense 1982 and 1991 solar activity and the onsets of the intense 2-3 kHz radio emission events detected by Voyager 1 and 2 in 1983-84 and 1992-93. This figure is adapted from Gurnett (1995)

In looking for possible sources, it soon discovered that the onset of the 1992-93 radio emission occurred approximately 419 days after the beginning of a series of strong coronal mass ejections and solar flares that occurred from May 25 to June 15, 1991. This solar activity produced the strongest terrestrial Forbush decrease in the cosmic ray intensity ever observed, even up to the present time, $30\%$. Forbush decreases were first identified in the 1930s and are now known to be associated with large global-scale solar mass ejections and shock waves propagating outward through the heliosphere from the Sun. The intense turbulent magnetic fields associated with these events inhibit the entry of galactic cosmic rays into the inner regions of the heliosphere. The consequences of the May-June 1991 solar events were subsequently detected by cosmic ray and magnetic field instruments not only at Earth but also at larger distances by four interplanetary spacecraft, Pioneers 10 and 11 and Voyagers 1 and 2, located at distances from the Sun ranging from 35 to 53 AU. Figure 2 shows some of the measurements made by these spacecraft (more details in VanAllen and Randall 1985; Webber et al. 1986; VanAllen and Fillius 1992; Webber and Lockwood 1993). The association of the 1992-93 radio emission event with these solar disturbances provided the first strong indication that the 2-3 kHz radio emissions were of heliospheric origin. This connection was soon greatly strengthened when it was discovered that the 1983-84 radio emission event occurred 412 days after the onset of another period of strong solar activity in mid-1982. The mid-1982 activity produced the second strongest terrestrial Forbush decrease in the cosmic ray intensity ever observed, $\sim 21\%$. The resulting time-delay relationship is illustrated in Fig. 3, which shows the two strong Forbush decreases, A and B, and the two strong 2-3 kHz radio emission events, A’ and B’, on comparable time scales. The heliospheric origin of the radio emission was strengthened even further when a series of *V1* spacecraft rolls designed to calibrate the magnetometer allowed PWS radio direction-finding measurements. These measurements showed that the source of the 2-3 kHz radio emission was beyond but near the HP.

Motivated by the solid cause-effect relationship implied by Fig. 3, Gurnett et al. (1993) proposed that the intense radio emission events were triggered when outward propagating global shock waves and ejected solar plasma reached and interacted with the HP. The upward drifting frequency components of the radio emission were attributed to the shocks propagating through a region of increasing plasma density immediately beyond the HP, called a “density ramp”. It was further proposed that the radio emissions were generated by mode conversion from electron plasma oscillations stimulated by upstream electron beams generated by the outward propagating shocks. This mechanism was essentially patterned after the mechanism via which type II radio emissions are produced by shock waves propagating through density gradients in the solar corona. Such shock waves are known to produce radio emission at the electron plasma frequency $f_{p}$ and its harmonic 2$f_{p}$ (Wild et al. 1954). Subsequent studies have since shown that the primary 2-3 kHz radio emission occurs at the fundamental, $f_{p}$ (Cairns et al. 2003; Mitchell et al. 2004). The velocity of the shock wave ejected from the Sun can be determined from the arrival times of the shock at the various spacecraft. Moreover, since the travel time of the shock wave from the Sun to the HP was roughly 415 days, it was possible to compute the distance of the HP for the first time. The only significant complication was that the shock propagation speed, 600 to 800 km/s, was only known in the inner region of the heliosphere ($< 53$ AU), and that the shock would almost certainly propagate slower in the inner heliosheath (IHS). Furthermore, the ratio of the distance to the termination shock to the distance to the HP was poorly known. Gurnett et al. (1993) estimated that the distance from the Sun to the HP was between 116 to 177 AU. Later, using more refined parameters and simulations that were explicitly designed for the May-June 1991 series of solar events, Steinolfson and Gurnett (1995) estimated the distance to the HP to be 128 AU (156 AU) for a shock with the speed of 600 km/s (800 km/s). For comparison, some twenty years later, *V1* and *V2* crossed the HP at ∼120 AU (Stone et al. 2013; Burlaga et al. 2018b), a distance in reasonable agreement with the above estimates, given the uncertainties involved.

**Fig. 4:**
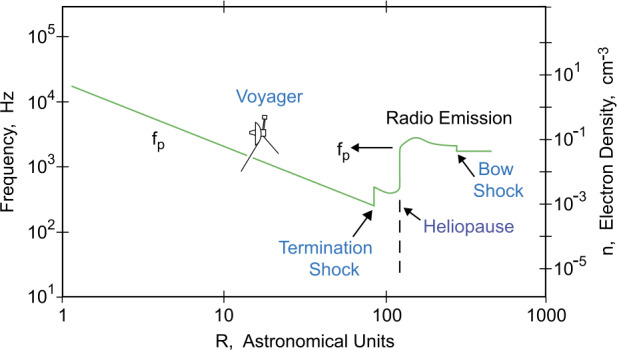
A simple model for the radial distance, $R$, variation of the electron plasma frequency, $f_{p}$. The model assumes that the 2-3 kHz radio emission is produced at or near $f_{p}$ by mode conversion from locally generated electron plasma oscillations upstream of shocks propagating outward from the Sun, comparable to the mechanism responsible for Type II solar radio bursts. This figure is adapted from Gurnett (1995)

Electron plasma oscillations are a characteristic oscillation of electrons in a plasma. The frequency of the oscillations is related to the electron density via the equation $f_{p} = 8980 \sqrt{n_{e}}$ Hz, where $n_{e}$ is the electron density in cm^−3^ (Gurnett and Bhattacharjee 2017). Before reaching the HTS, the plasma flow is supersonic, and the electron density in the nearly spherically symmetric plasma surrounding the Sun must vary approximately as $n_{e}$ $\sim 1/R^{2}$, where R is the radial distance from the Sun. It follows from the square root dependence in the equation for $f_{p}$ that the electron plasma frequency must then vary as 1/R till reaching the HTS (Fig. 4). Since the plasma density can only increase by a factor of four at a strong shock (Gurnett and Bhattacharjee 2017), it follows that the plasma frequency can increase by only a factor of two at the HTS. This can be even less for the weaker HTS mediated by suprathermal pickup ion (PUI) that *V2* finally observed (Richardson et al. 2008).

As shown in Fig. 4, once the spacecraft crosses the HTS, the plasma frequency is expected to decrease slower than 1/R because the subsonic plasma no longer undergoes a symmetric radial expansion but rather is deflected downstream toward the tail of the heliosphere. Contrary to intuition, at the HP the plasma frequency must increase dramatically. This increase is due to pressure balance across the HP, which dictates that the plasma density must increase significantly as the spacecraft passes from the hot, $\sim 10^{5}-10^{6}$ K, heliospheric plasma into the relatively cool, $\sim 10^{4}$ K, interstellar plasma. This leads to a corresponding abrupt increase in the plasma frequency at the HP by almost a factor of ten, as shown in Fig. 4. Although the magnetic field also contributes somewhat to the pressure, the plasma density and temperature play the primary role in controlling the pressure balance across the HP. Since the time delays and shock propagation speeds implied by the 2-3 kHz radio emission events dictate that the radio source must be at a distance well over 100 AU from the Sun, the plasma frequency in the region immediately inside the HP must be correspondingly low, only a few hundred Hz, much too low to account for heliospheric 2-3 kHz radio emission. Thus, of the two apparent locations for the radio emission source, the HTS and the HP, the only possible location for the source must be near or beyond the HP where the plasma frequency is high enough to be in the 2-3 kHz range.

## The VLISM Plasma Density

**Fig. 5:**
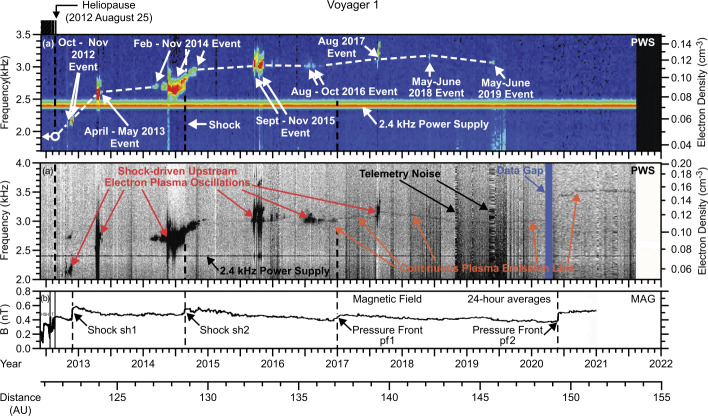
The top panel shows a color frequency-time spectrogram of all the relatively intense shock-driven electron plasma oscillations detected by the *V1* PWS. The middle panel shows a more sensitive gray-scale frequency-time spectrogram that show the much weaker nearly continuous plasma emission line detected by the *V1* PWS. Density scales are given for both on the right. The bottom panel shows the corresponding magnetic field detected by the *V1* magnetometer. Only two of the intense shock-driven electron plasma oscillation events can be directly associated with locally observed shocks. The others are believed to be due to electron beams arriving from distant shocks along interstellar magnetic field lines

The previous section showed how the knowledge of plasma density in the VLISM has been used to estimate the source of observed freely-propagating radio emissions in the 80s and 90s. This section focuses on the VLISM plasma density in more detail and discusses the density variation with respect to the distance from the HP. We present different theories that suggest explanations about the density change in front of the HP. As can be seen in the top panel of Fig. 5, after the *V1* HP crossing in 2012, a series of electron plasma oscillation events occurred over the subsequent several years, each of which is currently believed to be associated with a shock wave propagating outward from the Sun (more details in the following sections). To date, eight such plasma oscillation events have been identified in the *V1* PWS waveform data, two of which have been directly associated with shocks identified locally at the spacecraft by the jump in the magnetic field (see Sect. 4.1 for details). The plasma oscillation events not associated with locally observed shocks are thought to be generated by electron beams streaming along interstellar magnetic field lines from distant shocks. According to a theoretical model (Cairns and Zank 2002; Cairns et al. 2003; Gurnett et al. 2021b), the plasma oscillations are produced by electrons from the shock that form a beam streaming outward along interstellar magnetic field lines ahead of the shock in a region known as the “electron foreshock”. The geometry is comparable to the electron foreshock observed ahead of planetary bow shocks (Filbert and Kellogg 1979; Cairns 2011).

The middle panel of Fig. 5 shows a more sensitive gray-scale frequency-time spectrogram that reveals the much weaker nearly continuous plasma emission line recently detected by the *V1* PWS (Ocker et al. 2021). As can be seen from the electron density scale on the right-hand side of both top panels, the electron density has increased considerably since the spacecraft crossed the HP. The rate at which the density has increased is especially large in the first two years after the HP crossing, a region that corresponds to the “density ramp” invoked by Gurnett et al. (1993) to explain the upward frequency drift of the 2-3 kHz radio emission. This region of increasing plasma density immediately beyond the HP is called the Heliospheric Boundary Layer (HBL) by Pogorelov et al. (2017). This nomenclature recalls the term commonly used to describe the “boundary layer” that forms in the flow of fluids around solid objects, but the physical mechanisms involved here are quite different. Pogorelov et al. (2017) model showed that the width of the HBL, about 40-50 AU, is comparable with the p-H charge exchange mean free path. Moreover, exploiting an analogy with the hydrodynamic problem of a blunt body in supersonic motion, Pogorelov et al. (2021) pointed out that the plasma density should not be expected to reach its maximum at the HP surface, except perhaps on the stagnation streamline.

**Fig. 6:**
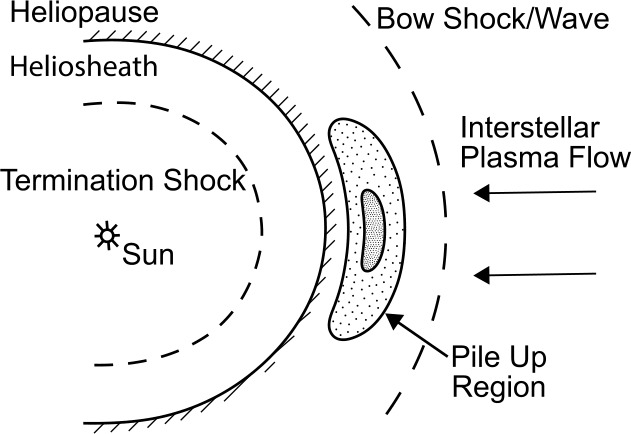
The plasma density “pile up” region that is postulated to exist upstream of the heliosphere based on the gas-dynamic flow simulations by Steinolfson (1994). This model implies that as *V1* proceeds further outward from the Sun the plasma density will increase, reach a peak, and eventually decline before reaching the undisturbed pristine ISM

The physical reason for forming the boundary layer around the HP has been the subject of much debate, and several interpretations have been offered. Fluid dynamic simulations by Steinolfson (1994) showed the occurrence of a region of increased plasma density immediately ahead of the HP, as indicated by the shaded area in Fig. 6. Gurnett et al. (1993) interpreted this density increase as being due to the “pile up” of plasma in the region immediately upstream of the HP. In the context of fluid flow, this pile up effect, also sometimes called the “snowplow” effect, usually implies a significant level of viscosity in the fluid due to molecular collisions. According to this interpretation, the density should eventually peak as *V1* moves farther from the Sun and then decline somewhat as it moves into the pristine ISM. So far, the Voyagers have not reached this peak, and it is currently not known if it will ever be reached. The low-resolution simulation of Steinolfson (1994) cannot properly capture and distinguish the plasma density increases across the HP, and it continues increase with a smaller gradient afterward. Higher-resolution simulations of Pogorelov et al. (2017) managed to distinguish these two increases.

It is known experimentally that the flow reaches its maximum entropy at the stagnation point. On the other hand, the gas density reaches a maximum between the bow shock and the HP. Unfortunately, there are no direct measurements of entropy on the surface of the HP. The distance from the HP at which the plasma density reaches its maximum is affected by the shape of the HP and the properties of the LISM. It so happens that the width of the HBL is similar, but not equal, to that of the hydrogen wall. On another theory, Gurnett et al. (1993) mentioned that the interaction of the interstellar plasma with the neutral “hydrogen wall” described by Baranov (1990) and Baranov and Malama (1993) may play a role in controlling the radial density profile in the region near HP.

Another model shows that pile up at the upwind HP forms a plasma depletion layer (PDL) through a combination of MHD and kinetic processes (Anderson and Fuselier 1993; Fuselier and Cairns 2013; Cairns and Fuselier 2017). This PDL is analogous to the one that forms upstream of the Earth’s magnetopause and other planetary magnetopauses (Gershman et al. 2013; Masters et al. 2014) despite the fact that the plasma conditions in the interstellar medium are significantly different from those in the inner heliosphere. As the magnetic field piles up against the HP, the magnetic field strength increases, and the temperature anisotropy ($T_{\perp}/T_{\parallel}$) of both the ions and the electrons increases. At some point, the temperature anisotropies will exceed the threshold for the electromagnetic ion cyclotron (EMIC) instability and the equivalent instability for the electrons. EMIC waves grow rapidly and, through wave-particle interactions, pitch-angle scatter ions from the perpendicular to the parallel direction and reduce the temperature anisotropy below the wave-growth threshold. Ions that have a high parallel velocity leave the system, and as the magnetic field compresses further, the anisotropy again increases above the EMIC instability threshold, and the process repeats itself. Generally, oppositely-directed gradients in density and magnetic field strength are the standard signature for a PDL and evidence for such features exist in the V1 data (Cairns and Fuselier 2017).

**Fig. 7:**
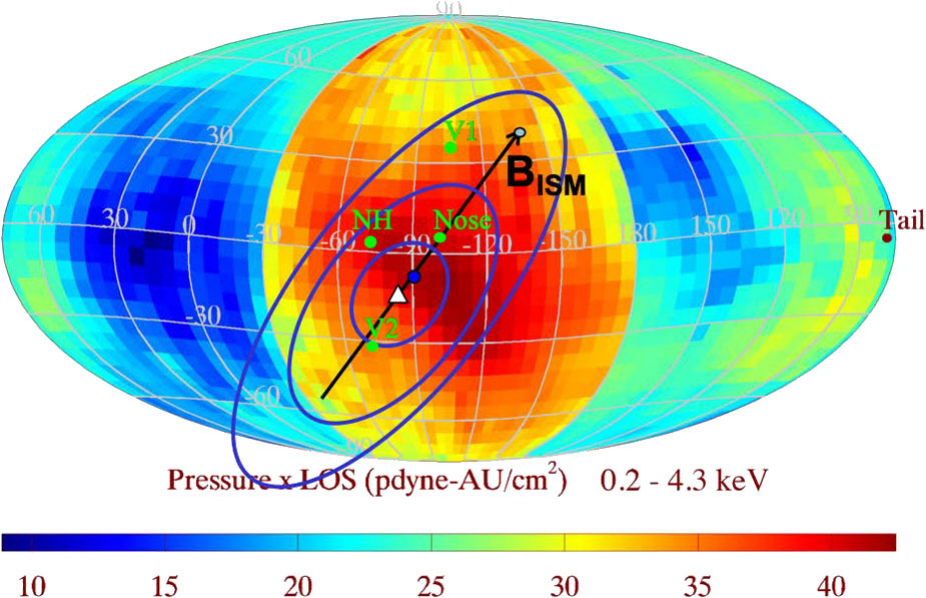
Predicted location and strength of the PDL at the HP (blue contours) superposed onto a sky map of the pressure × LOS from the IBEX ENA observations. This sky map is looking outward from the Sun. The maximum magnetic pressure on the heliosphere along the interstellar magnetic field direction that contains the nose direction is identified by the white triangle. Contours of the PDL surround this maximum pressure location. The green dots show the *V1* and *V2* and the New Horizons (NH) directions. The *V2* crossing of the HP is closest to the maximum in the PDL. Adapted from Cairns and Fuselier (2017)

Figure 7 shows the predicted location and strength of the PDL on a Mollweide-projected sky map. The sky map shows the pressure times line-of-sight (LOS) distance derived from IBEX energetic neutral atom (ENA) observations from 0.2 to 4.3 keV (see Schwadron et al. 2014). The point on the HP where the magnetic field draping is maximum occurs along the interstellar magnetic field direction at a point $\sim 40^{\circ}$ from the nose direction, indicated by the white triangle (Cairns and Fuselier 2017). Comparing the PDL contours with the HP crossing locations of *V1* and *V2*, it is clear that *V2* is much closer to the strongest part of the PDL than *V1* (see Cairns and Fuselier 2017). When *V2* crossed the HP in late 2018, it observed a much stronger magnetic field and a lower density than observed at the *V1* crossing (Burlaga et al. 2019; Gurnett and Kurth 2019; Fuselier et al. 2020) and thus stronger PDL.

While some basic properties of the PDL upwind of the HP are known from observations, much is unknown. The structure of the PDL depends on the properties of the VLISM adjacent to the HP. At the HP, the thickness of the PDL is of the order of 2-5 AU, and Coulomb collisions could mediate the temperature anisotropy, not allow the plasma instabilities to grow, and therefore reduce or eliminate the PDL formation process. Using a nominal plasma convection speed in the VLISM, Cairns and Fuselier (2018) showed that electrons are collision-dominated, and it is not clear how or if the properties of the PDL are affected by having collision-dominated electrons. Moreover, the PDL at the HP has interplanetary shocks propagating through it regularly. These shocks modify the plasma in the PDL and lead to time variability of the magnetic field strength in the layer (Cairns and Fuselier 2017). Such large-scale, frequent modifications to the density and magnetic field structures in the VLISM, as well as associated shock heating, may modify the PDL formation process in ways that are currently not known. Finally, magnetic reconnection at the HP mitigates the effects of magnetic field draping at the HP. Reconnection is a complicated process because of the complex draping of the magnetic field at the HP and the highly asymmetric plasma parameters across the boundary. On the other hand, Pogorelov et al. (2017) noticed a layer of depressed plasma density exists on the LISM side of the HP in simulations even without magnetic field (Baranov and Malama 1993) or in the absence of interstellar neutrals (Pogorelov and Matsuda 1998). Moreover, the density observed by Voyager is still increasing and it is not in the order of 3-12 AU as predicted by PDL model. Therefore, Pogorelov et al. (2017) argued that the PDL may be a tiny layer at the bottom of the HBL.

## Shocks in the VLISM

Partially transmitted heliospheric shocks at the HP are observed in the VLISM by *V1* (Gurnett et al. 2013b). Here, we first present the *V1* observations of shocks and pressure fronts in the VLISM (Sect. 4.1). In Sect. 4.2, we show different models that explain the propagation of the shocks to the outer heliosphere and the VLISM. We then focus on the observed VLISM shock in 2012 by *V1*, which appeared to be very weak and have a considerably large thickness (4.3). We present a theory to describe the 2012 VLISM shock and its collisionality to explain its wide thickness. Finally, a brief summary of the turbulence at the VLISM shocks is presented in Sect. 4.4.

### Voyager Observations

**Fig. 8:**
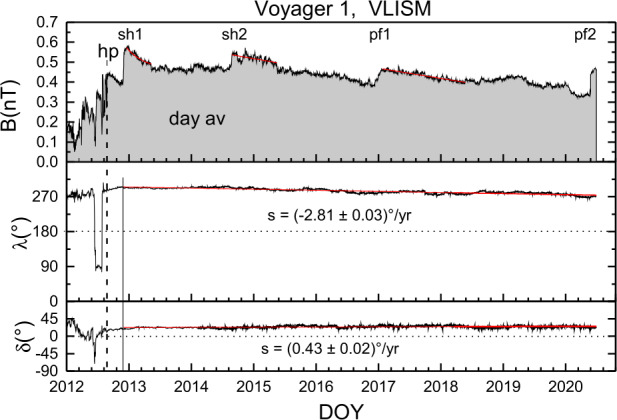
This figure shows the most recent structure of the magnetic field observed in the VLISM by *V1*. The magnetic field strength shows four notable structures corresponding to the largest local maxima in B in the top panel, namely 2 shocks (sh1 and sh2) as well as to “pressure fronts” (pf1 and pf2). The lower panels show a linear decrease in the azimuthal angle and a linear increase in the elevation angle with increasing time and thus distance from the sun, with no notable fluctuations observable on this scale. Adapted from Burlaga et al. (2021)

We present the *V1* observations of shocks and pressure fronts in the VLISM. Figure 8 shows a plot of *V1* observations of the magnetic field strength $B$, the azimuthal direction $\lambda $, and the elevation angle $\delta $ in a spacecraft-centered RTN coordinate system on a scale of ∼10 years (Burlaga et al. 2021). The magnetic field direction varied linearly with increasing time in the VLISM, as shown in the lower panels in Fig. 8. The labels in the top panel refer to shocks “sh1” and “sh2” and pressure fronts “pf1” and “pf2”. Each of them is described in the following subsections.

#### Shock 1 (sh1)

**Fig. 9:**
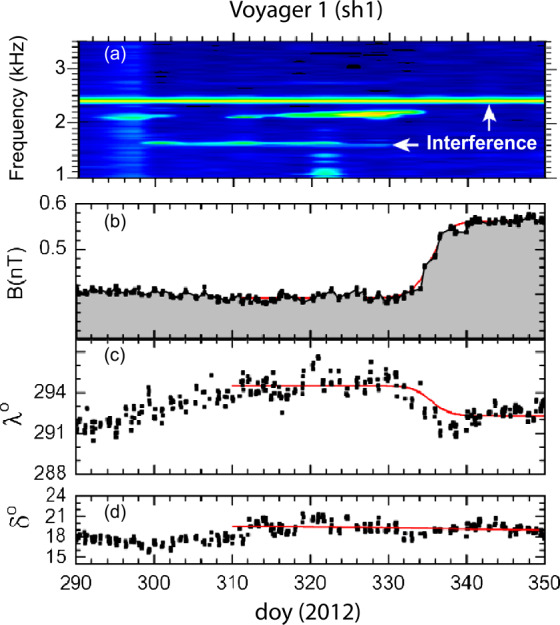
Panel (**a**) shows plasma oscillations near 2 kHz that were generated by energetic electrons accelerated by a shock shown by a large increase in $B$ near day 335. (**b**) The azimuthal angle of the magnetic field, $\lambda $, changed by a few degrees across the shock (**c**), but the elevation angle $\delta $ was nearly constant across the shock (**d**). This figure is from Burlaga et al. (2013a)

The presence of the first shock “sh1” in the VLISM was identified by Burlaga et al. (2013a) and Gurnett et al. (2013a), who observed in situ plasma oscillations that were possibly generated by electrons accelerated by a shock. The shock was then identified by an increase in $B$, as shown in Fig. 9. An increase in $B$ was observed by *V1* around day 330, 2012, which is at the end of the period containing electron plasma oscillations. The magnetic field strength profile shown by the red curve was obtained from a fit to $B(t)$ with a sigmoid function, which gives the asymptotic values $B_{1}$ = $0.392 \pm 0.001$ nT and $B_{2}$ = $0.562 \pm 0.002$ nT, and the inflection point (the center of the jump) occurred at time t = $335.43 \pm 0.07$ days. The small jump in $B$ ($B_{2}$/$B_{1}$ = 1.4) was shown by Burlaga et al. (2013a) to be consistent with a weak MHD shock. A small change ($\sim 2^{\circ}$) in the azimuthal angle $\lambda $ also occurred during this interval, with no associated change in $\delta $ (Figs. 9 c and d). The “width”, (i.e., shock ramp crossing time, $\tau $) is 1.23 days, and 80% of the change in $B$ occurred within a time of 4.4 $\tau $ = 5.4 days. The thickness of observed shock by *V1* in the VLISM is much larger (about 10^4^ times) than the expected thickness of an interplanetary shock in the solar wind at 1 AU. An explanation was published by Mostafavi and Zank (2018b,a), which is discussed later in Sect. 4.3.

The frequency of the electron plasma oscillations ahead of the jump in B was $f$ = 8980 Hz, which corresponds to an electron density of 0.05 cm^−3^. The shock normal (0.89, 0.40, 0.24) was obtained from the co-planarity theorem (Colburn and Sonett 1966) using the RTN components of the magnetic field, due to the unavailability of plasma velocity data at *V1*. The angle between the shock normal, $n$, and the radial direction was $28^{\circ}$, indicating that the shock was propagating close to the radial direction. The angle between n and the upstream magnetic field direction $B_{1}$ was $85^{\circ}$, showing that the shock was a quasi-perpendicular shock. From Fig. 9, it is evident that the magnetic field varied smoothly across the shock, indicating that the shock was laminar. Various parameters, including the shock speed of 40 km/s, were calculated by Burlaga et al. (2013a). The shock was demonstrated to be a laminar subcritical resistive shock (as opposed to dispersive), according to the Fredericks diagram (Kennel et al. 1985). If one adopts the terminology used to describe collisionless shocks in the SW (Kennel et al. 1985; Mellott 1985; Tsurutani and Stone 1985), the observations are consistent with the observations of a subcritical, low $\beta $, laminar, resistive, quasi-perpendicular shock.

#### Shock 2 (sh2)

*V1* observed a second shock in the VLISM on 2014/236 (2014.6438), which is labeled “sh2” in Fig. 8. This shock, like sh1, was associated with an electron plasma oscillation event described previously (Gurnett et al. 2013a; Burlaga et al. 2013a), which was presumably associated with electrons accelerated by the shock. These electron plasma oscillations at both shocks support the interpretation of the jumps in B as shocks. Here, we briefly discuss the properties of the sh2 event, with more details given in Burlaga and Ness (2016).

**Fig. 10:**
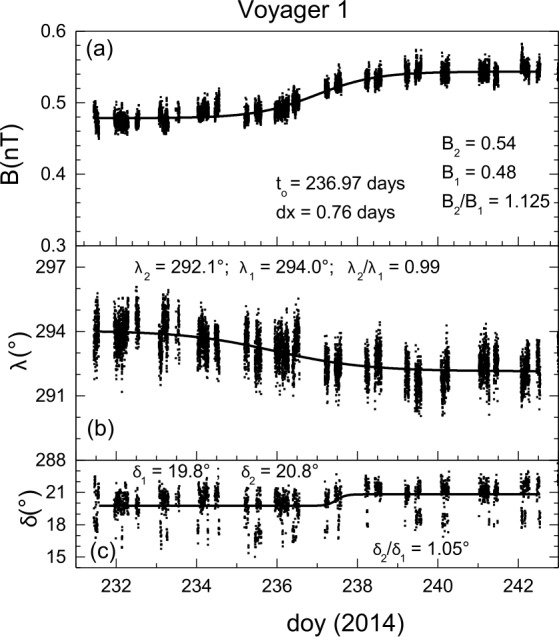
The structure of a shock observed by *V1* during 2014. The figure shows temporal variations of 48 s averages of B (**a**), $\lambda $ (**b**), and $\delta $ (**c**) as the shock moved past the spacecraft. Adapted from Burlaga and Ness (2016)

Figure 10 shows 48 s averages of B observed by *V1* during the interval from 2014, day 229 to 243. The sigmoid fit to the 2014 shock gives the asymptotic values $B_{1}$ = 0.48 nT, $B_{2}$ = 0.54 nT, hence $B_{2}$/$B_{1}$ = 1.125, the time of the inflection point to = 236.9723 days, and a “width” $\tau $ = 0.76 days. The passage time is defined as eighty percent of the change in $B$ and occurred within a time 4.4 $\tau $ = 3.3 days. The magnetic fields in both two observed shocks (sh1 and sh2) were unlike the highly variable magnetic fields observed at the HTS by *V2*. The sh2 was “laminar”, as can be seen in Fig. 10 that the transition across the shock was smooth. The shock normal is estimated from the co-planarity theorem to be (0.96, 0.54, -0.27). Thus, the corresponding angle between the shock normal and the radial direction was $16^{\circ}$, indicating that the shock was propagating nearly radially. The angle between $B_{1}$ and $n$ was $61^{\circ}$ consistent with a quasi-perpendicular shock. (Burlaga et al. 2008). The jump in $B$ and density across the sh2 were 1.125 and 1.11, respectively. A similar density jump is consistent with the prediction for a perpendicular shock.

The thickness of the 2014 shock is $\sim 10^{7}$ km which is about $10^{4}$ $d_{i}$ ($d_{i}=c/\omega _{pi}\approx 800$ km is the ion inertial length). The thickness of the 2012 shock was also $\sim 10^{4}\,d_{i}$ as mentioned previously. In comparison, the thickness of the ramp of the supercritical HTS observed by *V2* was of the order of 6000 km $\sim d_{i}$, and the thickness of the ramp and overshoot combined to make a shock thickness of the order of 25 $d_{i}$ (Burlaga et al. 2008). The thickness of a laminar subcritical resistive shock at 1 AU is about 3 $d_{i}$ (Greenstadt et al. 1975). Therefore, the observed shocks in the VLISM are ∼3000 times thicker than the observed subcritical resistive shocks at 1 AU and ∼500 times thicker than the supercritical HTS.

#### Pressure Front pf1

**Fig. 11:**
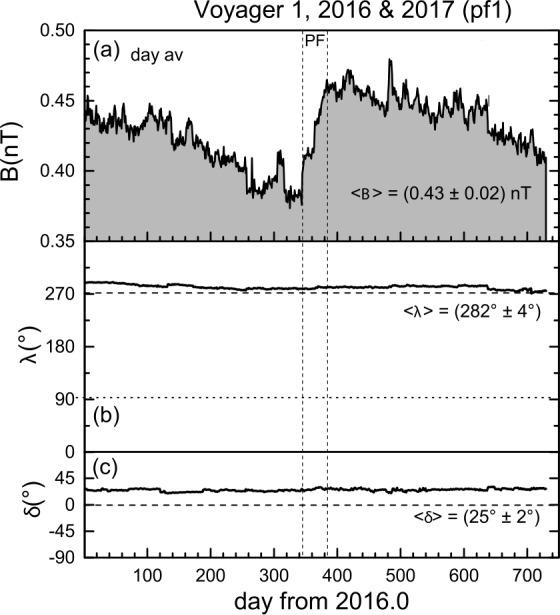
A pressure front (pf1), denoted by the vertical dashed line was observed by *V1* as an increase in B near day 346, 2016 (**a**). The pressure front was followed by a further long and slow decrease in $B$. The direction of $B$ remained nearly constant throughout the interval, as shown in panels (**b**) and (**c**). Figure from Burlaga et al. (2019)

*V1*, moving in the VLISM, observed a large magnetic feature characterized by a rapid increase in the daily averages of B beginning on 2016 day 346, rising to a local maximum on day 382, and declining nearly slowly until day 720 of 2016.0 (Fig. 11). The two vertical dashed lines show an interval in which the magnetic field and the magnetic pressure increased. This region is called a “pressure front, pf” and is discussed in detail by Burlaga et al. (2019). The magnetic field strength increased across the pressure front by a factor of $B_{2}$/$B_{1}$ = 1.19, from $B_{1} = 0.39 \pm 0.03$ nT to $B_{2} = 0.46 \pm 0.03$ nT and the passage time was estimated to be ∼35 days as indicated by the two vertical dashed lines. The Boltzmann fit gives a width parameter $w = 7.6$ days and thus $80\%$ of the passage time is $\tau = 4.4 \times w = 33.4$ days. This is in good agreement with 35 days obtained by visual inspection of the data. The inflection point of the Boltzmann fit, which is midway between the two vertical dashed lines, is at day 363.87. Following the pressure front, $B$ decreased more or less linearly until at least day 700 of 2016. Therefore, it is possible that pf1 was not a shock, since the passage time of ∼35 days is much larger than that of earlier shocks in the VLISM (details in Burlaga et al. 2018b). Nevertheless, the pressure front might steepen and evolve into a shock over time, whose characteristics might be determined by heat conduction (Baranov and Zaitsev 1998) and collisionality (Mostafavi and Zank 2018b,a).

Fraternale and Pogorelov (2021) reconsidered *pf1* and estimated that the spatial thickness may be about 0.25 AU (only twice the thickness of *sh1*), if the propagation speed is $\sim 30$ km s^−1^ in the fixed frame. This is the speed of the predicted pf1 in the Kim et al. (2017) data-driven model (see Fig. 14, where interstellar shocks slow down with increasing distance from the HP. Moreover, from a correlation analysis of the components of $\mathbf{B}$ Fraternale and Pogorelov (2021) suggested the possibility that *pf1* might have a two-step structure. The observation of two jumps has been ascribed to systematic errors in Burlaga et al. (2019). Although the presence of two compression waves following each other remains uncertain, we note that these shock interactions are abundant in numerical simulations (see Fig. 14 and animations in Pogorelov et al. (2021)).

#### Pressure Front pf2

**Fig. 12:**
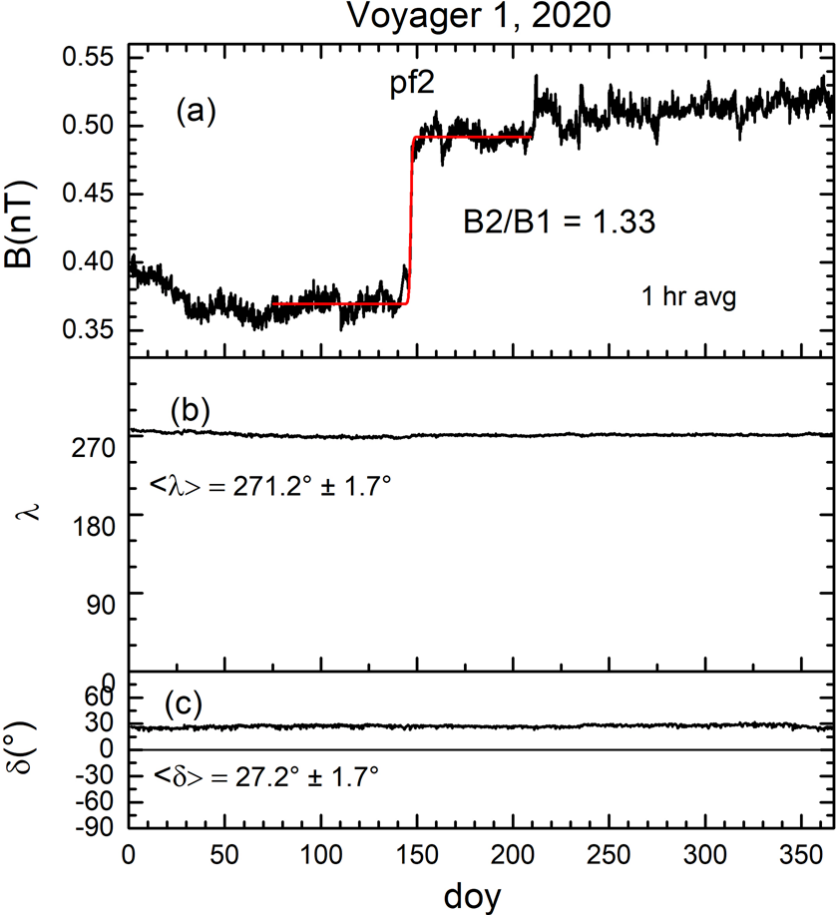
The second pressure front, pf2, observed by *V1* during 2020 as an abrupt increase in B by a factor of 1.33. There was no change in the direction of the magnetic field, as shown in panels (**b**) and (**c**). Adapted from Burlaga et al. (2021)

The second pressure front in the VLISM, pf2, is shown in Fig. 12. It shows that the magnetic field direction was nearly constant across the jump and throughout the year, with $\langle \lambda \rangle = 271.2^{\circ}\pm 1.7^{\circ}$ and $\langle \delta \rangle = 27.2^{\circ}\pm 1.7^{\circ}$, which are surprisingly close to the Parker spiral field directions in the heliosphere and heliosheath (Parker 1958) and is still the subject of discussion (see Kleimann et al. of this volume). The magnetic field strength shows that the most prominent feature of the VLISM magnetic field during 2020 is the large abrupt increase in $B$.

**Fig. 13:**
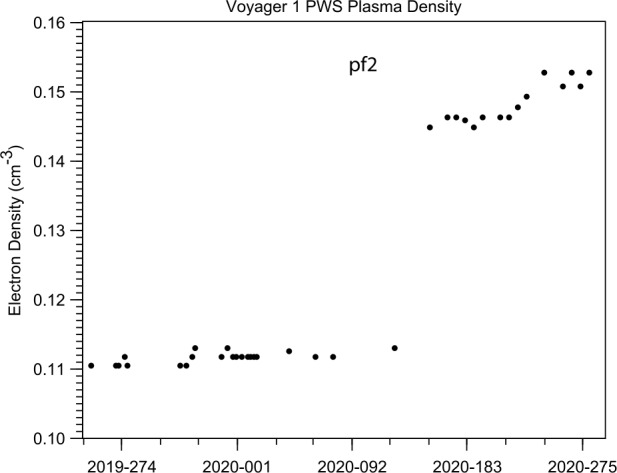
The electron density increased at the same time and by nearly the same amount as the magnetic field at the time that the pressure front pf2 moved past *V1* during 2020. Figure from Burlaga et al. (2021)

The jump size in $B$ was obtained by choosing the start time for the fit on day 76 and the end time on day 208. The sigmoidal fit (i.e., the red curve in Fig. 12) gives the ratio of $B_{2}$/$B_{1}$ to be about 1.33. A jump in density accompanied the jump in $B$, as shown by Burlaga et al. (2021), who determined the density using a new technique developed by Ocker et al. (2021) and Gurnett et al. (2021). They found a new line at the plasma frequency in the wave signal measured by the PWS experiment on *V1*, beginning in 2016 and continuing to the end of 2020. Unlike the waves at the plasma frequency associated with electrons driven by occasional shocks, the new line at the plasma frequency was presumably generated continuously by thermal processes. The density of VLISM plasma can be computed directly from the “thermal” plasma frequency, which is proportional to the square root of density. In this way, Burlaga et al. (2021) showed the presence of a large increase in the plasma density at the time that pf2 moved past *V1*, as shown in Fig. 13.

The interval during which the jump pf2 took place was ∼11.0 days, which is the “passage time” of the jump, based on the sigmoid fit to $B$. As mentioned earlier, the passage time of sh1 and sh2 was 5.41 days and 3.34 days, respectively, which are relatively close to the passage time of pf2 (about a factor of 2 difference). Burlaga et al. (2021) showed that pf2 was not associated with plasma oscillations driven by electrons presumably accelerated at a distant shock, unlike shocks sh1 and sh2. They showed no unusual intermittency or change in intermittency at pf2, which is consistent with the absence of a shock. However, there is no definitive proof showing that pf2 was moving supersonically or not. If pf2 is not a shock, it will likely evolve to a shock over some distance in the VLISM. The passage time of pf1 was ∼35 days, observed at 137.2 AU and the passage time of pf2 was 11 days at 149.3 AU. The shorter passage time at pf2 might be the result of the steepening of the pressure front as it moved to larger distances at later times.

### Shock Propagation

This section discusses the propagation and evolution of the solar wind structures to large distances from the Sun and their transit through the heliospheric boundaries into the VLISM. Having discussed the observations of shocks and pressure fronts in the VLISM, it’s natural to ask about the origin of these structures. They are thought to be associated with the large-scale solar wind disturbances formed near the Sun (coronal mass ejections and corotating stream interaction regions). However, their origin cannot be derived by tracing back the *Voyager* observations because of the memory loss of the disturbances as a result of interactions within the significant distance (Burlaga 1983). The observations in the VLISM must be computed from initial conditions close to the Sun. Transient and corotating flows evolve as they propagate, carrying magnetic fields and momentum from the Sun. Multiple solar wind transients can merge to form a large region with relatively strong magnetic fields, a Global Merged Interaction Region (GMIR) with a radial dimension ∼20 AU which can encircle the Sun and extends to high latitudes (Burlaga et al. 1984). The individual shocks produced by transient flows from the Sun can overtake and merge with the corotating shocks associated with corotating stream interaction regions within 10 AU tending to develop even stronger shocks, offsetting the decrease in shock strength associated with increasing distance from the Sun (Burlaga 1995).

Liu et al. (2014) used a 1-D MHD simulation and showed that the observed shock and radio emissions in the VLISM can be the consequence of a series of the CMEs in March 2012. Recently, Guo et al. (2021) extended their 1-D numerical MHD model by using inputs from OMNI, STEREO-A, and B at 1 AU and found that each of the plasma oscillations and shock events in the VLISM are linked to large-scale solar wind events. An observational study by Richardson et al. (2017) proposed that the five GMIRs observed by *V2* in the inner heliosheath may drive the shock waves observed by *V1* in the VLISM.

Whang and Burlaga (1994) published a 1-D model that shows how a shock driven by a GMIR interacts with the HTS, propagates through the heliosheath, and interacts with the HP to produce a shock in the VLISM. Interaction of the HTS with various types of solar wind disturbances have been studied in different models (e.g., Barnes 1993; Naidu and Barnes 1994; Steinolfson 1994; Pogorelov 1995; Story and Zank 1995, 1997; Baranov et al. 1996; Ratkiewicz et al. 1996; Zank and Müller 2003; Baranov et al. 2004; Pogorelov and Zank 2005; Washimi et al. 2007, 2011; Borovikov et al. 2012; Provornikova et al. 2013; Fermo et al. 2015). These studies considered the propagation of a shock wave, a contact discontinuity, a pair of forward-reverse shocks, and pressure pulses through the HTS. They described discontinuities and wave modes downstream of the HTS in the inner heliosheath and their interaction with the HP. For example, Zank and Müller (2003) explicitly showed the propagation of different disturbances into the VLISM using a 2D model that included neutral H. Borovikov et al. (2012) analyzed the interaction of small-amplitude waves generated in the heliosheath by corotating interaction regions (CIRs) in a 3D heliosphere in the presence of neutral atoms, and the interstellar and heliospheric magnetic field.

The ideal magnetohydrodynamic (MHD) interaction of a heliospheric shock and HTS is a complex problem. In two dimensions, the problem is determined by five parameters (Baranov et al. 1996): the Mach numbers of the HTS and the incident shock, plasma beta, angle between normal of the HTS and the shock, and the inclination of the heliospheric magnetic field to the HTS front. In 3D, the problem becomes even more complicated because these parameters may vary along the shock front, and shocks may not be steady. For example, for typical parameters for the HTS and a heliospheric parallel shock near 90 AU, an analytical theory (Baranov et al. 1996) predicts that a result of the interaction between the forward shock and HTS will be an altered forward shock propagating in the inner heliosheath with trailing contact discontinuity and a slow shock. 3D MHD simulations predict a very similar structure of the flow in the inner heliosheath (Provornikova et al. 2013). Washimi et al. (2007, 2011) considered effects of dynamic pressure pulses in the solar wind on the motion of the HTS and flow in the inner heliosheath in a 3D MHD model with boundary conditions taken from in-situ *V2* observations. They showed propagation of fast magnetosonic waves downstream the HTS, the motion of the HTS in response to the pressure pulses from upstream, and waves from downstream the shock. After reaching the HP, the shock causes the HP to move outward and propagates further into the VLISM. The motion also creates a fast magnetosonic wave reflecting from HP, which propagates toward the HTS. The shock in the VLISM is significantly weaker than the original heliospheric shock because of the interaction with HTS and HP and expansion through the heliosheath.

**Fig. 14:**
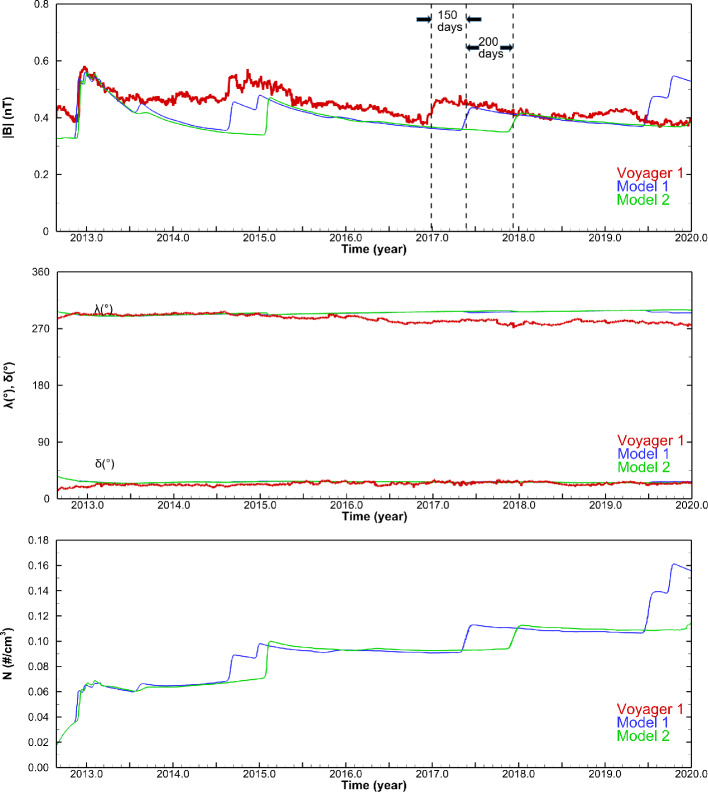
magnitude of magnetic field ∣B∣, the azimuthal, $\lambda $, elevation angles, $\delta $, and proton number densities for Model 1 (including all OMNI data) and Model 2 (ICMEs excluded from OMNI data) are shown in blue and green, respectively. The daily averaged *V1* MAG data are shown in red. Adapted from Kim et al. (2017)

**Fig. 15:**
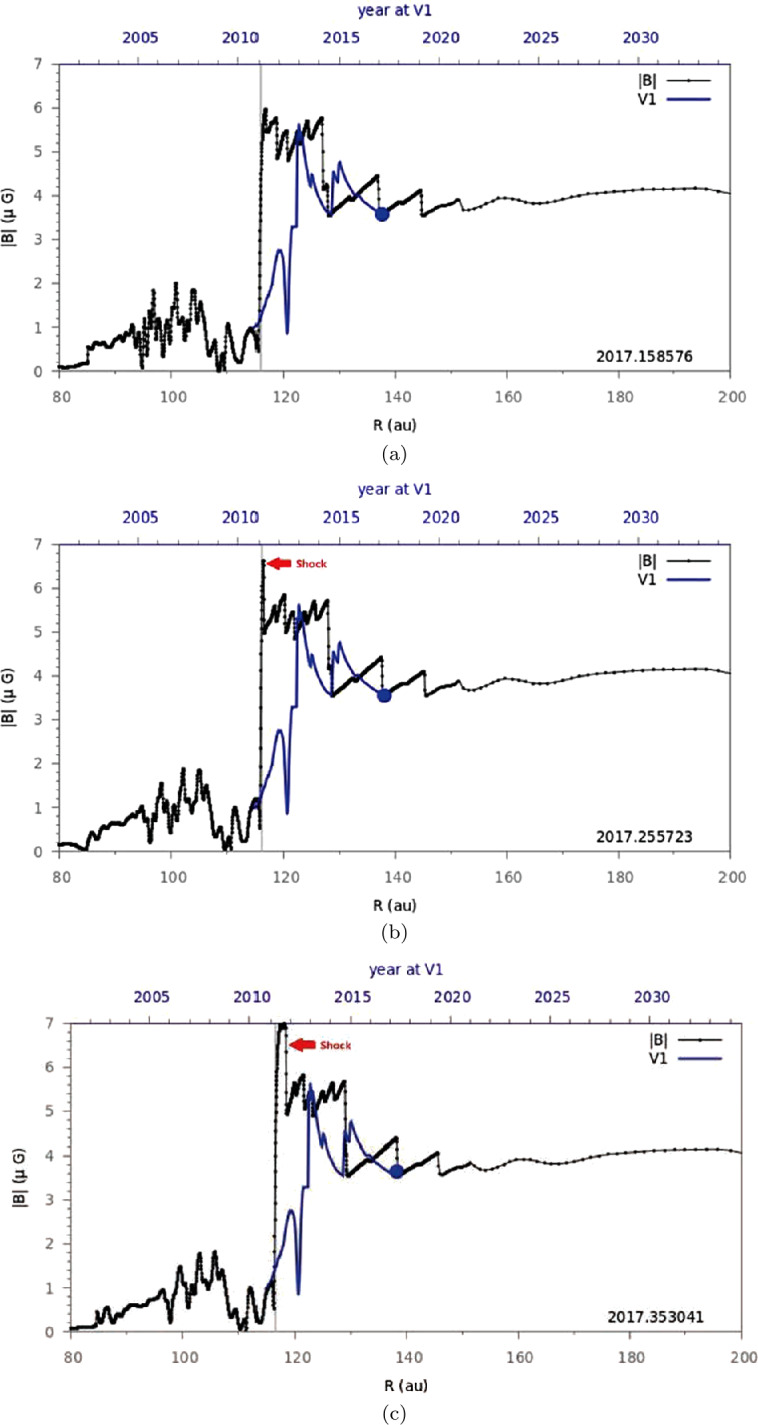
Transmission of a shock wave across the HP (marked by a gray vertical line) around 2017 DOY 94 in the Sun-*V1* direction. Panels (**a**)-(**c**) show the simulated magnetic field in the Sun-*V1* radial direction (black) on 2017 DOY 58, DOY 94, and DOY 129, respectively. For reference, the simulated magnetic field along the Voyager trajectory is also shown as a function of time in blue. Adapted from Pogorelov et al. (2021)

Assuming that all major shocks and compression waves observed in situ by *V1* in the VLISM are driven by solar activity, 3D data-driven time-dependent models can predict the arrival of disturbances and corresponding radio emissions to *V1* in the VLISM with reasonable accuracy. For example, Kim et al. (2017) extended the model and reproduced the first two shocks in the VLISM with only a slight difference in the arrival times (i.e., within ten days). On the other hand, their prediction of pressure fronts in mid-2017 and mid-2019 deviated from the actual arrival of compression waves by a wider margin (∼150 and ∼300 days, respectively). However, it is essential to account for the uncertainty of such data-driven predictions, though this was not explicitly discussed in the original paper. Figure 14 showed an alternative prediction (in green) where all ICME data in the OMNI dataset were removed at the simulation inner boundary. The results demonstrate not only the effects of ICMEs (or absence of ICMEs) on the shock arrival times at *V1* in the VLISM but also the upper bounds of the shock/compression wave arrival time window in the extremely unlikely case that no ICMEs at all were directed toward *V1*. In this hypothetical case, the shock/compression wave arrival times at *V1* were delayed by approximately 10, 160, 200, and 290 days, in chronological order. The original prediction (in blue) used only ICME data obtained in near-Earth space, so it is also possible that more (i.e., in number and/or intensity) ICMEs than implied by OMNI data may have been directed toward *V1* at certain times. Kim et al. (2017) estimated the arrival time window for shocks/compression waves to *V1* based on their model. Hence, the shocks and pressure front observed by *V1* on 2012 DOY 335, 2014 DOY 236, and 2016 DOY 346 – 2017 DOY 15 fall within the uncertainty windows of the shock arrival times predicted by Kim et al. (2017) at 2012 DOY 326 (±10 days), 2014 DOY 240 (±160 days), and 2017 DOY 145 (±200 days), respectively. These comparisons imply that for the first two shocks, the impact of *V1*-directed ICMEs was close to that of the Earth-directed counterparts, whereas for the 2017 structure, the former may have been significantly higher than the latter, thereby resulting in the late arrival of the simulated shock (Model 1 in Fig. 14) at *V1* by 150 days. Meanwhile, the most recent arrival of a pressure wave around 2020 DOY 147 falls just outside the prediction window of 2019 DOY 184 (±290 days). This discrepancy may be attributed to a possible error in the latitude extent of OMNI data at the simulation inner boundary since the SW structures driving this wave most likely originated at the simulation inner boundary in late 2015 when *V1* was on the fringe of the latitude extent of OMNI data. Consequently, a large-amplitude shock transmitted across the HP around 2017 DOY 94 as shown in Fig. 15 and reached *V1* in mid-2019 in the simulation (Pogorelov et al. 2021). With a slightly smaller latitude extent of OMNI, the merged interaction regions formed during this period would have been smaller in size and intensity and thus driving a more modest (and slower) shock/compression wave toward *V1* than originally predicted.

**Fig. 16:**
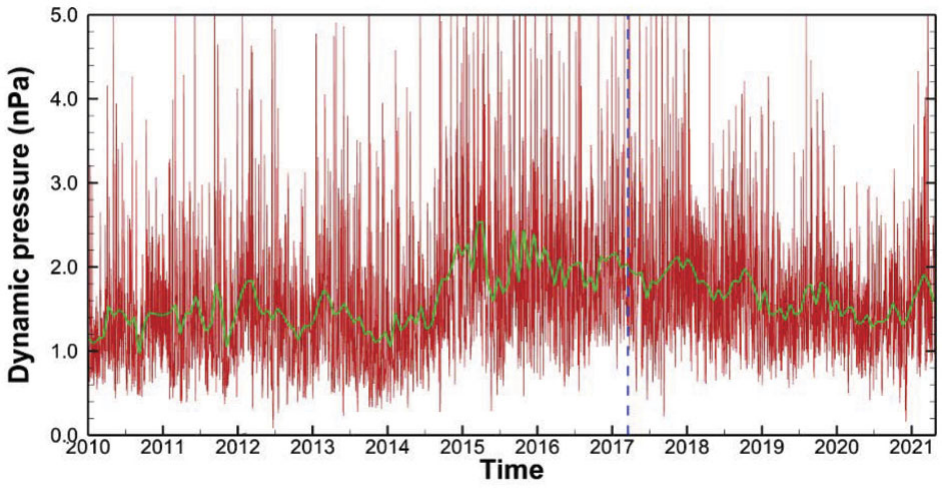
SW dynamic pressure at Earth between 2010 and 2021. Daily averages are shown in red, and 25-day boxcar averages are shown in green. The vertical blue dashed line marks the time of the last boundary update of the Kim et al. (2017) model. Daily averages of OMNI data (King and Papitashvili 2005) are shown in red

It is also important to notice the steady decline of the SW dynamic pressure since early 2017 (see Fig. 16), when the original model of Kim et al. (2017) stopped updating the inner boundary conditions (marked by a blue vertical dashed line). Considering that the response of the HP to changes in the SW at 1 AU is typically delayed by 1-2 years, the HP could have moved in closer to the Sun in 2018 and 2019, thus increasing the wave propagation distance (and also time) to *V1* in the VLISM. While data-driven models can be useful for short-term predictions, any predictions beyond two years should be taken with caution, due to the unsteady, ever-changing SW.

### Collisionality of the VLISM

As discussed in Sect. 4.1, the observed shock by *V1* in 2012, sh1, is remarkable in its substantial width, being about $10^{4}$ times broader than a shock with similar properties observed close to Earth. *V1* with a speed of about 17 $\text{km}$ $\mbox{s}^{-1}$ passed the 2012 VLISM shock in about 8.7 days. Our theoretical understanding of collisionless shocks is not capable of explaining such a broad shock structure.

Mostafavi and Zank (2018b) presented a theory to describe the VLISM and the structure of observed shocks. Interstellar neutral atoms, thermal protons and electrons, and nonthermal PUIs are the primary components of the VLISM. The charge-exchange time scale of neutral hydrogens and protons is sufficiently large that their coupling can be neglected in the content of VLISM shocks. The collisional mean free paths of PUIs and thermal VLISM protons and electrons are very large. This leads to a PUI population that is not equilibrated with background thermal VLISM plasma within about $75- 100$ AU of the HP (Zank et al. 2014). PUIs experience pitch-angle scattering due to magnetic field fluctuations, thus introducing dissipation terms such as collisionless heat flux and viscosity.

One can calculate the collisional streaming timescale of a charged particle “$a$” colliding with a stationary Maxwellian background population of charged particles “$b$” by (for more details, see Zank (2015))

1 $$ (\tau ^{ab}_{s})^{-1} = \frac{n_{b} q_{a}^{2} q_{b}^{2} \ln\Lambda}{2 \pi \epsilon _{0}^{2} m_{a}^{2} V_{Ta}^{3}} \frac{m_{b}}{m_{a}} (1+\frac{m_{a}}{m_{b}}) \frac{T_{a}}{T_{b}} \frac{G(x_{b})}{x_{a}}, $$

2 $$ G(x) \equiv \frac{f(x)-x f'(x)}{2x^{2}}, $$

where the $m$, $n$, $T$, and, $q$ denote mass, number density, temperature, and charge of each particles, respectively. Here, $\ln \Lambda $ is the Coulomb logarithm, $V_{T}$ is the thermal speed, and $\epsilon _{0}$ is the vacuum permittivity. $G(x)$ is the Chandrasekhar function, $f(x)$ is the error function, and $x_{a/b} \equiv v/V_{Ta/b}$.

**Fig. 17:**
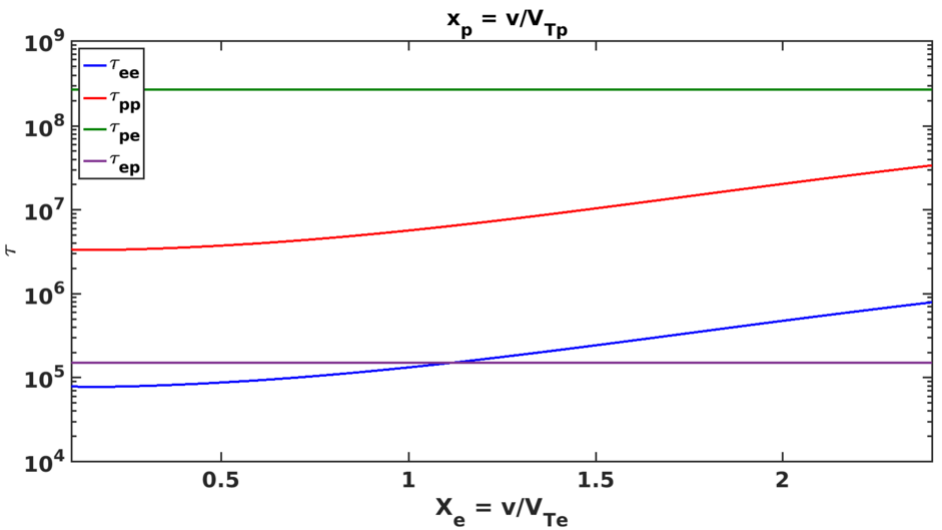
The $ee$, $pp$, $pe$, and $ep$ collisional time-scales (in seconds) for the thermal VLISM plasma as a function of velocity. Note that these collisional time scales only apply on the thermal gas particles and not to PUIs. This figure is adapted from Mostafavi and Zank (2018b)

McComas et al. (2015) combined IBEX/Ulysses observations and concluded that the VLISM electrons and protons are equilibrated and have a temperature of about 7000–9500 K. The VLISM with a temperature of 7500 K has protons and electrons with thermal speeds of 11 and 477 km s^−1^, respectively. The electron plasma oscillations observed by the plasma wave instrument on *V1* indicated an electron density of about 0.06 cm^−3^ in the vicinity of the 2012 shock (Gurnett et al. 2013a, 2014). The magnetic field magnitude measured by *V1*’s magnetometer upstream of the 2012 VLISM shock was about 0.38 nT (Burlaga et al. 2013a). Mostafavi and Zank (2018b) used these parameters to calculate the collisional timescales, $\tau $, and collisional mean free paths, $\lambda $, for electron-proton ($ep$), proton-electron ($pe$), proton-proton ($pp$), and electron-electron ($ee$) collisions. Figure 17 shows the collisional timescales for the thermal VLISM plasma as a function of $x$. Although the time-scales for $pe$ and $ep$ collisions are independent of velocity, the $pp$ and $ee$ collisional time-scales depend on velocity and are not constant. The calculated collisional timescales and mean free paths upstream of the 2012 VLISM shock are (Mostafavi and Zank 2018b)

3 $$\begin{aligned} &\tau ^{ep}=1.5\times 10^{5}~\mbox{s},\ \lambda ^{ep}=0.47~\text{AU}; \quad \tau ^{pe}=2.7 \times 10^{8}~\mbox{s},\ \lambda ^{pe}=20.4~\text{AU}, \end{aligned}$$

4 $$\begin{aligned} &\tau ^{pp}=4.2\times 10^{6}~\mbox{s},\ \lambda ^{pp}=0.31~\text{AU}; \quad \tau ^{ee}=7.5 \times 10^{4}~\mbox{s},\ \lambda ^{ee}=0.24~\text{AU}. \end{aligned}$$

The collisional mean free paths are small, and thus the VLISM is collisional with respect to the thermal gas, unlike the collisionless heliosphere. Coulomb collisions introduce dissipation terms such as collisional thermal heat conduction and viscosity in an MHD description of the VLISM. Electrons do not produce a large amount of heat conduction or viscosity compared to protons due to their small mass. The proton-proton collisional mean free path is very small compared to that of proton-electron collisions, and thus is the dominant collisional term in the VLISM.

Mostafavi and Zank (2018a,b) studied the 2012 VLISM shock structure with a two-fluid model. They treated PUIs as a separate pressure component, $P_{I}$, in the system since they are not equilibrated with the background thermal gas (Zank et al. 2014). Proton-proton collisions dominate and introduce a collisional dissipation into an MHD description of the VLISM. Using the parameters upstream of the VLISM shock, the derived diffusion coefficient and the thermal viscosity associated with proton-proton collisions are about $10^{15}~\mbox{m}^{2}\,\mbox{s}^{-1}$ and $2.5 \times 10^{-8}~\text{kg}\,\mbox{m}^{-1}\,\mbox{s}^{-1}$, respectively. Nonthermal PUIs in the VLISM are generated by secondary charge exchange and have a very small number density ($n_{p} \approx 5 \times 10^{-5}~\text{cm}^{-3}$) (Zirnstein et al. 2014). Their characteristic collisionless heat conduction and viscosity values in the VLISM are $\sim 10 ^{14}~\mbox{m}^{2}\,\mbox{s}^{-1}$ and $3.57 \times 10^{-13}~\text{kg}\,\mbox{m}^{-1}\,\mbox{s}^{-1}$, respectively (details in Mostafavi and Zank 2018b,a). The dissipation associated with thermal gas collisions is much greater than PUI collisionless dissipation. Mostafavi et al. (2017b,a) showed that the 2012 weak VLISM shock could not be mediated by the small dissipation associated with PUIs.

**Fig. 18:**
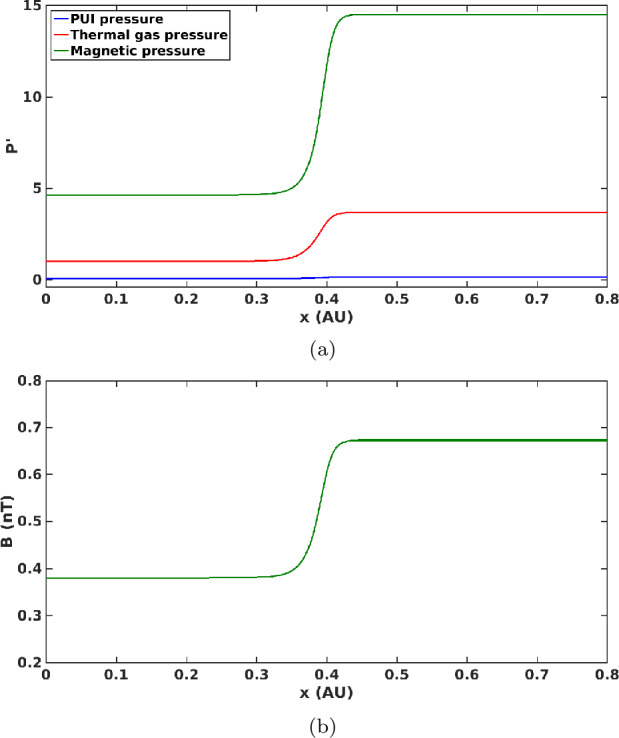
(a): The normalized PUI, thermal gas, and magnetic pressures to the thermal gas pressure far upstream of the shock as a function of distance (au). The shock is dominated by the thermal gas and magnetic pressures and PUIs only contribute a very small percentage of the pressure through the shock. (b): The magnetic field (nT) through the shock shows a smooth and weak transition. The compression ratio is $\sim 1.67$. These plots are the results of numerical simulations presented by Mostafavi and Zank (2018b)

Upstream of the VLISM shock, the plasma beta is $\simeq 0.2 < 1$, the Alfvén and sound speeds are 34 $\mbox{km}$ $\mbox{s}^{-1}$ and 14 $\text{km}\,\mbox{s}^{-1}$, respectively. The shock speed should be less than 52 $\text{km}$ $\mbox{s}^{-1}$ in a frame in which the Sun is stationary (Mostafavi and Zank 2018b) to ensure it is a subcritical shock (Burlaga et al. 2013a). An MHD simulation of the 2012 shock propagation in the VLISM by Kim et al. (2017) suggested a shock speed of about 50 $\text{km}$ $\mbox{s}^{-1}$ in the stationary Sun frame. Mostafavi and Zank (2018b) used a shock speed of 40 $\text{km}$ $\mbox{s}^{-1}$ with respect to the stationary Sun and modeled the VLISM shock structure. Figure 18a shows the normalized PUI, thermal gas, and magnetic pressure as a function of distance through the VLISM shock. The dominant components upstream and downstream of the shock are the magnetic field and thermal gas pressures, and thus the thermal gas collisional heat flux and viscosity modify the shock structure. PUIs do not contribute a large percentage of the pressure both upstream and downstream of the shock. Unlike the HTS, PUIs behave almost adiabatically (i.e., $P_{I} \rho ^{-\gamma _{I}}$ = constant), while the thermal gas is exclusively heated by the weak VLISM shock. The weak VLISM shock, therefore, exhibits properties very different from the HTS. The change in magnetic field across the shock represents a smooth transition connecting the upstream to the downstream state with a compression ratio of $\sim 1.67$ (Fig. 18b). The shock thickness of $\sim 0.12$ AU or $\sim 8.7$ days corresponds to the collisional heat conduction length scale, which is consistent with the observed thickness by *V1*. The first surprising conclusion is that the weak VLISM shocks are mediated by proton-proton collisions and not by wave-particle interactions.

There are a number of questions that arise when VLISM shocks are considered. In the collisionless solar wind, shocks with marginally-critical Mach numbers can accelerate ions and excite low-frequency MHD wave precursors at frequencies as low as ∼0.01 of the gyrofrequency in the ion foreshock (e.g. Tsurutani et al. 1983). This typically occurs for quasi-parallel shocks. Only infrequently precursors were observed at quasi-perpendicular shocks (e.g. Kajdič et al. 2012; Gedalin et al. 2021). It is possible that backstreaming ions propagate along the magnetic field lines from less oblique parts of the shock, which may be as far as 10–20 AU from *V1* if $\theta _{Bn}\approx 65^{\circ}$, as estimated by Burlaga and Ness (2016). However, it is not clear if VLISM shocks, being weak and remarkably thick, can generate electron and ion beams. A possible suggested mechanism is magnetic mirroring (Wu 1984; Cairns et al. 2003; Mitchell et al. 2004; Jokipii and Kóta 2014), but this topic needs further investigations.

The above theory presented in this section assumed that thermal gas has a Maxwellian distribution with the temperature of ∼7500 K. PUIs represent a non-thermal separate component that is not equilibrated with the thermal plasma in the VLISM (within a distance of 75-100 au; see Zank et al. 2014). Based on the assumed temperature, the collisional mean free path for p-p is small (∼0.25 au), which leads to a collisional VLISM. On the other hand, there is another competing view about the collisionality of the VLISM. Fraternale and Pogorelov (2021) assumed that the thermal plasma in the VLISM near the HP is hotter and has a temperature of about 30,000 K, decreasing to 25,000 K 25 AU upstream of the HP. These values are motivated by recent observations in the VLISM by Richardson et al. (2019) and global model results (e.g., Kim et al. 2017). According to *V2* observations after the HP crossing, the thermal plasma temperature may be in the range between 30,000 K and 50,000 K, whereas models typically predict a VLISM temperature of 15,000–30,000 K within $\sim 50$ AU from the HP, essentially due to the plasma compression and ionization of interstellar neutrals. In this case, the p-p collisional mean free path would be ∼2–4 AU (see Table 1 in Fraternale and Pogorelov (2021)), which raises the question of either Coulomb collisions can fully mediate the observed VLISM shocks. However, understanding the shape of velocity distribution functions and the heating mechanisms of the VLISM plasma remains a challenge that demands further investigations. The observations reported by Richardson et al. (2019) are puzzling and difficult to interpret since it is unclear what the thermal source of the unexpectedly high temperature in the VLISM might be. Models of the global solar wind-LISM interaction do not provide a ready explanation for possible heating since the conflation of pickup ions and thermal plasma leads to a single temperature that does not reflect a non-equilibrated plasma. The challenges in accurately measuring and interpreting the plasma observations of the VLISM with the *V2* plasma instrument are not trivial, especially in the absence of a physical explanation for a much higher than expected temperature.

### Turbulence at VLISM Shocks

Shock waves propagating in turbulent neutral media or plasmas typically experience a strong mutual interaction with the turbulence, which can substantially modify the shock dynamics, spatial structure, and propagation properties (Andreopoulos et al. 2000). Shocks in collisionless plasma are peculiar in that they interact with both the preexisting *in situ* turbulence and with kinetic-scale wave activity generated in the foreshock region by the instability of energetic ion and electron beams backstreaming from the shock front (e.g. Tsurutani and Stone 1985; Stone and Tsurutani 1985; Eastwood et al. 2005; Trotta et al. 2021; Pitňa et al. 2021). Turbulence and shocks are ubiquitous in the heliosphere. There are indications that the VLISM turbulence in the boundary layer adjacent to the HP is significantly affected by the presence of the heliosphere (Zank et al. 2017, 2019; Matsukiyo et al. 2019; Fraternale and Pogorelov 2021). The reader is referred to Fraternale et al. in this volume for a review of the turbulence properties in the outer heliosphere and VLISM.

**Fig. 19:**
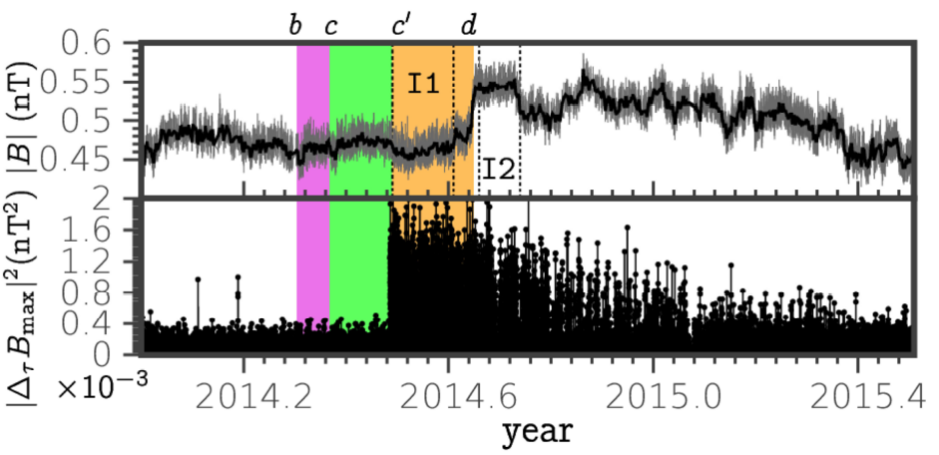
Shock wave observed in 2014 by *V1* in the VLISM (sh2). We highlight the region of enhanced GCR flux (purple) and the region of intense plasma waves observed by PWS (green). The orange band indicates the region of enhanced MHD fluctuations, also shown in the lower panel in terms of increments of the maximum variance component of $\mathbf{B}$. Labels *b*, *c*, and *d* denote the outer boundaries of the GCR and electron foreshocks as described by Gurnett et al. (2015), and the shock itself (DOY 111, 134, 236, respectively). Label $c'$ (DOY 178) indicates the region of enhanced magnetic field fluctuations. Reproduced from Fraternale et al. (2020)

As it was described in Sect. 4.1, the major shocks observed by *V1* in the VLISM, sh1 and sh2, are quasi-perpendicular, subcritical and laminar. Fraternale et al. (2019) analyzed high-resolution, 48 s averaged MAG data and suggested the possibility that VLISM turbulence may be mediated by shocks. More recently, Fraternale et al. (2020) focused on the second shock observed by *V1* (2014.65) and found a region with enhanced magnetic field turbulence starting from DOY 177, 2014, 44 days after *V1* encounters the region of intense and spiky plasma waves detected by PWS (Gurnett et al. 2015, 2020), and in correspondence of the isotropization of GCR flux. This enhanced turbulence region extends to the downstream region, until the beginning of 2015. Figure 19 shows the magnitude of $\mathbf{B}$ and the increments, $\Delta \mathbf{B}=\mathbf{B}(t)-\mathbf{B}(t+\tau _{b})$, of the maximum variance component. The event was interpreted in the context of the foreshock model (Gurnett et al. 2015, 2020), which includes the electron and cosmic-ray foreshock. The scale used for the increments, $\tau _{b}=144$ s, may correspond to a spatial scale of $\approx 6\,d_{i}$, under Taylor’s frozen-turbulence approximation. The turbulence spectrum was found to be amplified and steepened on frequencies in the range $2\times 10^{-4} \lesssim f \lesssim 10^{-2}$ Hz—spatial scales about $1000~\mathrm{km} \lesssim \ell _{\perp}\lesssim 10^{-3}~\mbox{AU}$ (1-200 $d_{i}$) in the direction perpendicular to $\mathbf{B}$. The observed regime then lies in the MHD-to-kinetic transitional range and may include kinetic effects related to ionized particles. Unfortunately, the kinetic regime (with respect to the thermal plasma) cannot be observed by *Voyagers*. The turbulent cascade is characterized by fine-scale structures of mixed transverse/compressible nature and compatible with a filamentary topology. Remarkably, the fluctuations’ amplitude (up to $\sim 0.1 $B) is such that small-scale intermittency could be revealed for the first time in the VLISM. The abruptness of the described event suggests that local processes responsible for the injection of wave power in the small-scale MHD regime exist in the VLISM. Shock-related kinetic processes are natural candidates, but a number of questions arise when the VLISM shocks are considered. It should be noted that among the four shock/compression waves observed so far by *V1*, the 2014.65 shock was the only one displaying enhanced turbulence in front of it (Burlaga et al. 2021). The reasons for this are still unknown, but it may be of interest to recall that the most intense plasma wave event so far was detected in front of this shock (Gurnett and Kurth 2019), and that the estimated angle between the shock-normal and the average $\mathbf{B}$ was smaller than at other shocks/compressions.

We have discussed above the presence of microscale turbulence. In principle, shocks can also interact with turbulent fluctuations on scales larger than the shock width, such as the compressible turbulence first observed by Burlaga et al. (2013b). Burlaga and Ness (2016) highlighted the presence of quasi-periodic fluctuations with the periodicity of $\approx 28$ days in the interval 2014.8 to 2015.4, behind the 2014.65 shock, suggesting their heliospheric origin. The spectral and correlation analysis in Fraternale and Pogorelov (2021) has emphasized the coexistence of turbulence and quasi-periodic fluctuations in different VLISM intervals between 2013 and 2020. They are weak with respect to the background field, but more intense in “disturbed” intervals behind shocks or compression waves. This suggests the possibility of interaction between VLISM turbulence and shocks for fluctuations at relatively large scales ($10^{-3} \lesssim \ell _{\perp}\lesssim 5$ AU). On the other hand, Zank et al. (2019) pointed out that models of small-amplitude waves/shock interaction predict that simple transmission of perturbations across a very weak shock cannot generate significant levels of MHD turbulence (McKenzie and Westphal 1968). Fraternale and Pogorelov (2021) estimated the turbulent Alfvén Mach number using the average magnetic field fluctuations, $M_{\mathrm{A,turb}}=\delta b_{\mathrm{rms}}/C_{\mathrm{A}}$ is approximately equal to 0.027. It can become as high as 0.1 for the largest perturbations. For instance, the average intensity of fluctuations of the magnetic pressure in 2014 is $\delta P_{\mathrm{m}}\approx 1.5\times 10^{-14}$ Pa, which is about 50% of the pressure jump across the shock. In this case, as suggested by numerical simulations, the interaction may induce a corrugation of the shock surface and modify the instantaneous structure and speed (e.g. Lele and Larsson 2009). Ultimately, from the VLISM turbulence perspective, it is interesting to note that steep power-law turbulence spectra with spectral index close to -2 at the largest scales observed by (Burlaga et al. 2018a; Fraternale and Pogorelov 2021) may be ascribed to a Burgers-like phenomenology where shocks and steepened compression waves coexist with the turbulence in the VLISM adjacent to the HP.

## The Heliospheric Bow Shock/Wave

A bow shock forms when a star travels with a supersonic velocity with respect to its interstellar space. Initially, the presence of a bow shock in front of our moving Sun through the LISM was widely accepted by many studies (for example, see Fahr et al. 2007; Zank et al. 2009, and references therein). However, McComas et al. (2012) analyzed IBEX observations and showed that the interstellar velocity is slower than the fast magnetosonic speed, and thus there should not be a bow shock ahead of the heliosphere. Zank et al. (2013) theoretically showed that the existence of secondary PUI population in the VLISM increases the sound speed in the VLISM, leading to further weakening or even yielding to a bow wave instead.

During the past few decades, several MHD models have investigated the existence of bow shock or wave. Solutions of the SW-LISM interaction simulations strongly depend on the choice of boundary conditions, both in the SW and in the LISM. For example, the choice of different interstellar magnetic field can have significant impact on the bow shock/wave properties such as its shape, size, and intensity (for example, see Kotlarz et al. 2018, and reference therein). However, the properties of the unperturbed LISM are not measured directly, and Voyager measurements of the VLISM are made in the region highly affected by the presence of the heliosphere. It is known (Izmodenov et al. 2005; Pogorelov and Zank 2006; Pogorelov et al. 2008, 2009; Zirnstein et al. 2016) that the average deflection of the neutral H atom flow in with respect to its direction in the unperturbed LISM occurs predominantly in the plane formed by the velocity and magnetic field vectors, $\mathbf{V}_{\infty}$ and $\mathbf{B}_{\infty}$, in the unperturbed LISM. *Ulysses* and *IBEX* observations of neutral He atoms make it possible to estimate $\mathbf{V}_{\infty}$, $\mathbf{B}_{\infty}$, and the LISM temperature $T_{\infty}$. However, these observations are based on certain assumptions regarding the distribution function of neutral He at the spacecraft. In particular, it is important to distinguish He atoms that experienced no charge exchange from those which did, the latter being called the “warm breeze.” The assumption of Maxwellian distribution for pristine He atoms arriving at their detectors is a clear simplification. As shown by Pogorelov et al. (2008), even the flow of H atoms which experienced no charge exchange is still deflected from its original direction. Their distribution function is modified due to preferential removal of certain H atoms, which did experience charge exchange. The same should be possible for He atoms. In addition, Swaczyna et al. (2021) showed that pristine He atoms could be heated in the VLISM by Coulomb collisions with ions.

The situation is complicated by heating of the LISM by secondary neutrals, especially by the neutral SW (NSW), which are born in the supersonic region but can propagate deep into the LISM, decelerating it and increasing its temperature (Gruntman 1982; Zank et al. 1996a). For this reason, parametric analysis is required to understand whether the bow shock exists and, especially, what its topology is. This is because fast magnetosonic waves’ propagation speed depends on the direction of their propagation with respect to the magnetic field direction. Florinski et al. (2004) showed that the bow shock could be a slow-mode shock if $B_{\infty}$ is large enough. However, this is not considered as a realistic scenario now, especially because it became clear that a fast-mode bow shock, if it exists for smaller $B_{\infty}$, simply becomes weaker and ultimately disappears, rather than changes its type. The problem with identifying a bow shock in numerical simulations is primarily technical and entirely depends on the numerical resolution. The application of adaptive mesh refinement (AMR) and Cartesian grids, which make it possible to maintain uniform resolution in all coordinate directions, made it possible to analyze the effect of changes in the LISM properties on the bow shock existence.

**Fig. 20:**
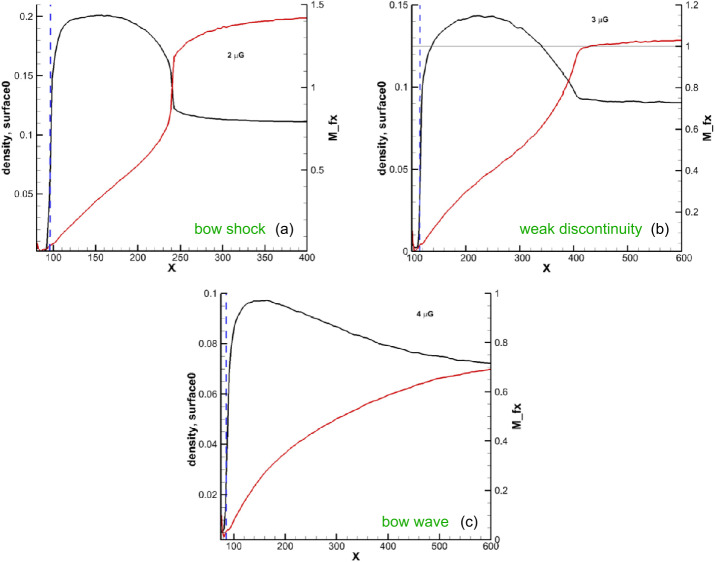
Distributions of the plasma density (black lines) and fast magnetosonic Mach number (red lines) along the $x$-axis in AU for different ISM magnetic fields. The vertical blue dashed lines show the HP position. The simulation results are from Pogorelov et al. (2017)

Zank et al. (2013) analyzed the MHD equations with charge exchange source terms and found that hot and fast neutral H created in the heliosphere charge exchange with ISM ions and mediate the bow shock. They showed that the charge exchange could effectively result in a flow that is analogous to a Laval nozzle, with charge exchange effectively creating a “throat” through which the super-fast magnetosonic flow could be decelerated smoothly to a sub-fast magnetosonic flow. In this way, a bow shock can be eliminated in much the same way as mass loading can eliminate a cometary bow shock (although the physical mechanism is slightly different, the principle is analogous). As a result, the shock transition can change from a super-fast magnetosonic to a sub-fast magnetosonic flow over a large distance (e.g., 200 AU; see equation ten and discussions in Zank et al. 2013, for more details). Figure 20 shows the distributions of plasma density and fast magnetosonic Mach number for increasing $B_{\infty}$. The 2, 3, and 4 $\mu $ G values $\mathbf{B}_{\infty}$ present cases that the flow is super-fast magnetosonic, weakly super-fast magnetosonic, and sub-fast magnetosonic far upwind the HP, respectively (Zank et al. 2013; Pogorelov et al. 2017). Notice that the other properties of the unperturbed LISM should also be modified, including the direction of $\mathbf{B}_{\infty}$, according to the table in Pogorelov et al. (2017).

## Summary and Open Questions

The large-scale disturbances generated by the Sun, such as CMEs, CIRs, and MIRs, are efficient drivers of shock waves in the heliosphere. These shocks first can propagate in the heliosphere, interact with and influence the heliosphere’s boundaries (i.e., HTS and HP), and then traverse into the VLISM. About 40 years ago, Voyagers’ observations of the strong radio emission showed the first evidence of a heliospheric shock propagating beyond the HP. *V1* crossed the HP and entered the VLISM in 2012 for the first time, and since then, multiple shock waves and pressure fronts with unanticipated structures have been observed. The properties of the VLISM shocks observed by *V1* are surprisingly different from shocks inside the heliosphere because these shocks are extremely broad and weak, and unlike the heliospheric collisionless shocks, thermal collisions dominate their structures. The origin and evolution of observed VLISM pressure fronts are not well understood via either modeling or theory. Generally, the VLISM observations showed that the very different physics of the VLISM affects the properties of turbulence, shocks, and pressure waves in this region.

Even though the Voyagers were not appropriately instrumented to investigate the outer heliosphere, HP, and the VLISM, they have identified and partially answered many fascinating questions about these regions while raising many new questions. Ultimately, to answer some of those old and new questions, we need a future dedicated and ideally-instrumented mission, an Interstellar Probe (McNutt et al. 2019), to venture further and make new discoveries of the nature of our home heliosphere within the interstellar medium. Other than future missions with proper comprehensive instruments, our theoretical understanding and modeling of the heliosphere and its interaction with the ISM need to be improved significantly. Some of the open questions that need to be addressed in future studies and future missions are

- How do PUIs, reconnection, and instabilities affect the structure of HP?
- How does the VLISM density change with distance from the HP to the pristine ISM? Does the density eventually roll-over to a somewhat lower value? What is the reason for the observed continuous increasing plasma density beyond the HP?
- What are the properties of the heliospheric shocks that persevere and reach the VLISM? What is the origin of pressure waves in the VLISM?
- What is the role of collisionality in the VLISM, and on what scales is the VLISM collisional?
- Is the plasma temperature inferred from *V2* observations as high as suggested, and if so, what is the additional heat source?
- For what physical processes is collisionality important, and do collisionless wave-particle effects become important or even dominant in the ISM?
- How do shocks in the VLISM contribute to turbulence in the VLISM and LISM?
- What are the properties of PUIs in the VLISM, based on their various origins? How far in the VLISM are nonthermal PUIs found? Do VLISM PUIs affect shock properties?
- How far is the interstellar medium mediated by the heliosphere, and where is the transition from VLISM to the pristine interstellar medium?
- How do very weak subcritical shocks in the VLISM accelerate electrons?
- Is there a bow shock or bow wave in front of the HP in the ISM? Where is its location?
- If a heliospheric bow shock exists, what is the nature of interactions between interplanetary shocks extending into the VLISM and the bow shock?
